# Gray matter blood flow and volume are reduced in association with white matter hyperintensity lesion burden: a cross-sectional MRI study

**DOI:** 10.3389/fnagi.2015.00131

**Published:** 2015-07-08

**Authors:** David E. Crane, Sandra E. Black, Anoop Ganda, David J. Mikulis, Sean M. Nestor, Manus J. Donahue, Bradley J. MacIntosh

**Affiliations:** ^1^Heart and Stroke Foundation Canadian Partnership for Stroke Recovery, Sunnybrook Research InstituteToronto, ON, Canada; ^2^Division of Neurology, Department of Medicine, University of TorontoToronto, ON, Canada; ^3^Brain Sciences Research Program, Sunnybrook Research Institute, University of TorontoToronto, ON, Canada; ^4^Department of Medical Imaging, The Toronto Western Hospital and the University of TorontoToronto, ON, Canada; ^5^Institute of Medical Sciences, University of TorontoToronto, ON, Canada; ^6^MD/PhD Program, Faculty of Medicine, University of TorontoToronto, ON, Canada; ^7^Departments of Radiology and Radiological Sciences, Psychiatry, and Physics and Astronomy, Vanderbilt UniversityNashville, TN, USA; ^8^Department of Medical Biophysics, University of TorontoToronto, ON, Canada

**Keywords:** arterial spin labeling, cerebral blood flow, hippocampus, small vessel disease, white matter hyperintensities, insula

## Abstract

Cerebral White Matter Hyperintensities (WMH) are associated with vascular risk factors and age-related cognitive decline. WMH have primarily been associated with global white matter and gray matter (GM) changes and less is known about regional effects in GM. The purpose of this study was to test for an association between WMH and two GM imaging measures: cerebral blood flow (CBF) and voxel-based morphometry (VBM). Twenty-six elderly adults with mild to severe WMH participated in this cross-sectional 3 Tesla magnetic resonance imaging (MRI) study. MRI measures of GM CBF and VBM were derived from arterial spin labeling (ASL) and T1-weighted images, respectively. Fluid-attenuated inversion recovery (FLAIR) images were used to quantify the WMH lesion burden (mL). GM CBF and VBM data were used as dependent variables. WMH lesion burden, age and sex were used in a regression model. Visual rating of WMH with the Fazekas method was used to compare the WMH lesion volume regression approach. WMH volume was normally distributed for this group (mean volume of 22.7 mL, range: 2.2–70.6 mL). CBF analysis revealed negative associations between WMH volume and CBF in the left anterior putamen, subcallosal, accumbens, anterior caudate, orbital frontal, anterior insula, and frontal pole (corrected *p* < 0.05). VBM analysis revealed negative associations between WMH and GM volume in lingual gyrus, intracalcarine, and bilateral hippocampus (corrected *p* < 0.05). The visual rating scale corroborated the regression findings (corrected *p* < 0.05). WMH lesion volume was associated with intra-group GM CBF and structural differences in this cohort of WMH adults with mild to severe lesion burden.

## Introduction

Cerebral small vessel disease (SVD) is associated with cognitive and functional deterioration (Jokinen et al., 2009; Kreisel et al., 2013), stroke and death (Poggesi et al., 2011), and is reportedly the most common brain disease (Thompson and Hakim, 2009). T2-weighted MRI can be used to visualize and quantify white matter hyperintensities (WMH) of presumed vascular origin, which represents a common SVD subtype (Wardlaw et al., 2013). WMH are thought to result from chronic diffuse subclinical ischemia that primarily impacts subcortical regions (Brickman et al., 2009; Makedonov et al., 2013b; Wardlaw et al., 2013). Although WMH are commonly observed in cerebral white matter, SVD-associated lesions can also occur in subcortical gray matter (GM) (Vermeer et al., 2007). Studies have reported negative associations between WMH lesion burden (henceforth referred to as WMH volume) and regional GM tissue volumes, with the majority of the evidence pointing toward reduced hippocampal volume (de Leeuw et al., 2006; Godin et al., 2009; Kloppenborg et al., 2012). The hippocampus is known to be affected by impaired perfusion resulting from cerebrovascular disease and/or systemic causes (Knoops et al., 2009). The use of structural MRI measures to study the impact of WMH on the brain has produced mixed results. One study, for example, found that WMH was negatively associated with global GM tissue volume without detecting a regional hippocampal volume reduction (Du et al., 2005). Another study reported significant WMH and structural associations when considering only WMH adults with the most extreme (i.e., upper quartile) phenotype (Eckerstrom et al., 2011).

Given the recent clinical availability of non-invasive CBF imaging using arterial spin labeling (ASL) MRI (Alsop et al., 2014), the purpose of the current study was to determine whether regional CBF differences could be explained by within group differences in WMH volume. CBF may be a useful neuroimaging marker because hemodynamic alterations may identify vulnerable brain regions not detected by structural imaging techniques. The literature shows that CBF is reduced in normal appearing white matter and WMH regions (O'Sullivan et al., 2002; Makedonov et al., 2013a) as well as global cortical and subcortical CBF regions that are remote from the WMH lesions (Bastos-Leite et al., 2008). The primary objective of this study was identify whether there are regions where absolute CBF is associated with WMH lesion volume, among older adults with a range in WMH volumes (i.e., mild to severe). We also use of high-resolution T1-weighted images to characterize the relationship between WMH and regional GM volume, based on a modified voxel-based morphometry (VBM) approach. We hypothesized that WMH volume will be associated with regional reductions in both GM CBF and VBM. Regression analysis was conducted using WMH volume as a continuous measure. In addition, two *post-hoc* tests were performed. First, a 6-point WMH visual rating scale, referred to as the Fazekas score or age-related white matter changes (Wahlund et al., 2001), was used to corroborate the regression findings. Second, we explored the extent to which the findings from the separate CBF and VBM analyses were inter-related by testing for partial correlations.

## Materials and methods

### Participants

Thirty participants were identified by chart review from a Cognitive Neurology clinic at Sunnybrook Health Sciences Centre. Participants were recruited based on the presence of radiological WMH findings. Inclusion criteria were based on a Fazekas rating greater than or equal to 2 out of a total of 6 points. Patients received a clinical assessment that included a detailed medical history and a Montreal Cognitive Assessment test (MoCA) (Nasreddine et al., 2005). The median MoCA score was 24.5 (range: 11–30), as assessed by a trained psychometrist. Exclusion criteria were: probable Alzheimer's disease diagnosis, history of overt stroke (cortical infarcts), unsafe for MRI, carotid dissection, known severe carotid stenosis, use of short-acting antihypertensives (e.g., calcium channel blockers), age < 50 years, and known genetic SVD, determined by testing for the notch 3 mutation in those with suspected CADASIL. Of the 30 participants included in the study, 2 were excluded based on probable AD pathology as determined by a neurologist, and 2 were excluded due to a visible cortical infarct. The remaining 26 study participants had mild to severe WMH, a history of transient ischemic attack (6), mild subcortical lacunes (2), and one had a history of severe diabetic hypoglycemia. Hypertension was defined by blood pressure measurement of systolic >140 mm Hg or diastolic >90 mm Hg, or use of antihypertensive medication. Cholesterol status was based on the use of a cholesterol-lowering agent. Exercise information was included because of its purported effects on cognition, as discussed in a recent review (Schmidt et al., 2013). This study was conducted with approval from the Sunnybrook Research Ethics Board. Informed consent was obtained from all participants prior to the study.

### Imaging protocol

MRI was performed on a 3 Tesla MRI system (Philips Achieva) using body coil transmission and an 8 channel receive head coil. Imaging sequences included: T1-weighted turbo-field-echo imaging (repetition time [TR]/echo time [TE]/inversion time [TI] = 9.5/2.3/1400 ms, 140 slices, flip angle = 8°, 256 × 164 matrix, 0.94 × 1.2 × 1.2 mm^3^ voxels, 8:56 min); FLAIR (TR/TE/TI = 9000/125/2800 ms, 52 slices, 240 × 217 matrix, 1 × 1.1 × 3 mm^3^ voxels, 4:48 min); CBF imaging with pseudo-continuous ASL with single shot echo planar imaging (TR/TE = 4000/9.7 ms, 18 slices, 64 × 64 matrix, 3 × 3 × 5 mm^3^ voxels, label offset = 80 mm, no background suppression, post-label delay/duration = 1600/1650 ms, 35 control and tag pairs, 2 dummy scan, 4:48 min) (van Osch et al., 2009), and a reference ASL scan for absolute quantification (TR/TE = 10,000/20 ms, 18 slices, 64 × 64 matrix, 3 × 3 × 5 mm^3^ voxels, 3 signal averages, with pre-scan: 0:40 min). Phase-contrast survey images were used to prescribe the labeling region 80 mm below the lowest ASL slice the labeling plane was perpendicular to the internal carotid arteries and in proximity to the C1 cervical vertebrae.

### Image processing

#### Brain segmentation

T1-weighted images were segmented into GM, WM, and CSF using the Semi-Automatic Brain Region Extraction (SABRE) tool (Ramirez et al., 2011). FLAIR images identified WMH vs. normal-appearing white matter with a C-means algorithm (Gibson et al., 2010). Total WMH volume was quantified as the volume of WMH after adjusting for head size by normalizing intracranial volume (ICV; where ICV = GM + WM + CSF) to an average head size of 1300 mL (Sanfilipo et al., 2004).

The Fazekas score was used to compare the WMH lesion volume regression approach. This was conducted independently by two experienced raters, blinded to clinical details, with a high inter-rater agreement (Pearson correlation coefficient, *r* = 0.98). Raters discussed the two discrepancies in the Fazekas score and agreed on the final values. Because deep and periventricular WMH were highly correlated, we combined them into a summed Fazekas score, with possible values ranging of 0–6. Lacunes of presumed vascular origin were identified by visual inspection by a trained investigator blinded to patient diagnosis and were defined as subcortical fluid-filled cavities (isointense with CSF) of 3–15 mm in diameter (Kloppenborg et al., 2012). For tissue segmentation, lacunes were counted as part of the CSF volume.

#### Cerebral blood flow

ASL data were analyzed using the FMRIB Software Library (FSL; fsl.fmrib.ox.ac.uk) and in-house software scripts. Co-registration of the ASL data was performed using MCFLIRT. Absolute CBF quantification was based on the following equation (van Osch et al., 2009):

(1) $$\text{CBF}=60\cdot 100\cdot \frac{\Delta \text{M}}{2\cdot \alpha \cdot {\text{T}}_{1,\text{b}}\cdot {\text{M}}_{0}}\cdot {\text{e}}^{\frac{\text{PLD}+\Delta {\text{t}}_{\text{z}}(\text{z}-1)}{{\text{T}}_{1,\text{b}}}}\cdot {\text{e}}^{\frac{\text{TE}}{{\text{T}}_{2,\text{t}}^{*}}}$$

where 60 provides units of s/min, 100 provides units of 100 g of tissue, ΔM is the ASL difference signal, α = 0.83 is the labeling efficiency, M_0_ is derived from the ASL reference image that incorporates the blood-brain partient coefficient, PLD = 1600 ms is the post-label delay, TE = 20 ms is the echo time of the ASL images, T1, b = 16,800 ms is the T1 of arterial blood, T^*^2, t = 60 ms the effective relaxation time of GM tissue (Peters et al., 2007). M_0_ was extrapolated from the ASL reference image by modeling exponential decay (i.e., 1/(1-exp(-TR/T1))) and was used in place of M_0, CSF_ to incorporate correction for image inhomogeneity. Tissue classification masks were transformed to ASL space to estimate CBF in GM and WM (GM-CBF and WM-CBF, respectively). A study-specific template was created with Advanced Normalization Tools (ANTs) and CBF images were warped to this template using non-linear registration then smoothed with a 4.6 mm full-width half max (FWHM) Gaussian kernel.

#### Voxel-based morphometry

Structural data were analyzed using VBM in FSL (Douaud et al., 2007), with the following steps. First, T1-weighted structural images were brain-extracted, regions identified as WMH, deduced on the basis of coregistered FLAIR image, were replaced with simulated normal-appearing WM values to prevent misclassification as GM. Images were GM-segmented and registered to the MNI 152 standard space using non-linear registration. The resulting images were averaged and flipped along the x-axis to create a left-right symmetric, study-specific GM template. Second, all native GM images were non-linearly registered to this study-specific template and modulated by the Jacobian determinant to correct for local expansion/contraction due to the non-linear component of the spatial transformation, then smoothed with a 4.6 mm FWHM Gaussian kernel.

### Statistics

Participant demographics were summarized as mean ± standard deviation (SD), median and range. The Kolmogorov-Smirnov test was performed on all measures to assess data normality. Voxel-wise group analysis was performed on CBF and VBM data to address the main hypothesis.

For CBF data, voxel-level absolute CBF estimates were correlated with WMH volume. The general linear model (GLM) test was performed on CBF voxels with an SNR > 1. The SNR image was created by taking the ratio of the mean image divided by the standard deviation image. For VBM, voxel-level GM estimates were correlated with WMH volume for all GM voxels. Age and sex were included as covariates in both voxel-wise analyses. Five thousand permutations were executed to characterize the null distribution for CBF and VBM datasets, separately. Significant clusters of voxels were identified using the threshold-free cluster enhancement with a *p* < 0.05, corrected for multiple comparisons (Smith and Nichols, 2009). Clusters were localized by non-linear transformation to a template brain (ICBM-152).

Two *post-hoc* tests were conducted to explore the findings provided by the independent analyses of WMH on CBF and VBM. The first *post-hoc* test used the Fazekas rating scale as a categorical variable. Mean CBF values and GM estimates from ROIs identified by the voxel-wise analysis were used as dependent variables in this analysis. In the second *post-hoc* test, we calculated the partial correlation between the mean CBF values and GM estimates in these ROIs, with and without WMH as a covariate to explore the inter-relationship between CBF and VBM techniques, and test for the shared variance between all three variables.

Two-tailed significance values were reported. Statistical analyses were performed with Matlab (Mathworks, Natick, MA) and SPSS 20.0 for Windows (IBM, Armonk, New York).

## Results

**Figure 1:**
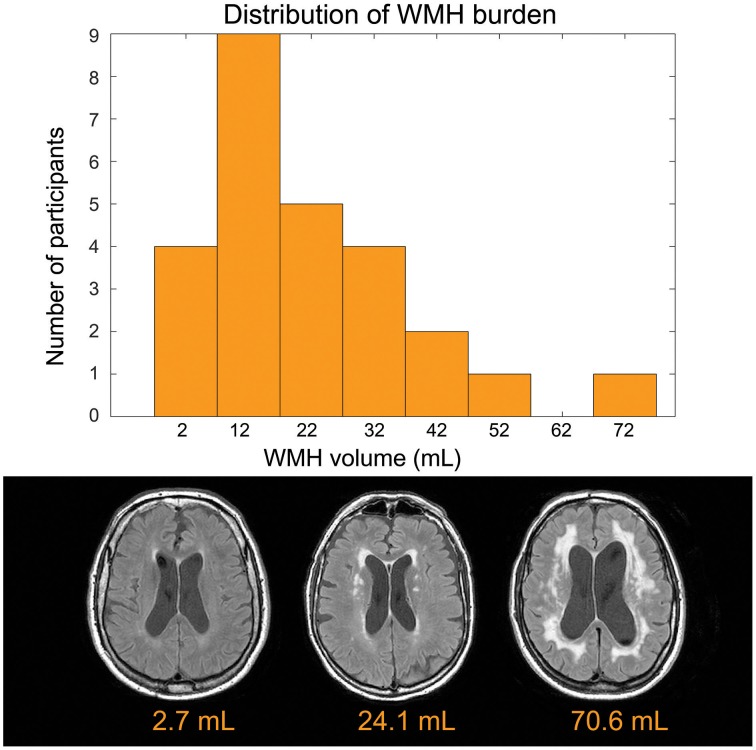
**Top:** Distribution of disease burden for this group. The white matter hyperintensity volume is adjusted to account for head size differences. **Bottom**: Representative FLAIR images.

The mean age was 73.3 ±8.8 years and 14 of 26 participants were women (see Table 1 for summary details). The distribution of WMH volumes did not differ significantly from normal (Figure 1), hence WMH volumes were used as the independent variables for the CBF and VBM voxel-wise analyses. Two participants had lacunes of presumed ischemic origin ranging in diameter from 7.5 to 8.0 mm.

**Table 1:** **Participant demographics (*n* = 26)**.

	**Mean (std)**	**Median**	**Range**
Age, years	73.3 (8.8)	73.5	51.0–87.8
Women	54%	–	–
MoCA	23.9 (4.6)^†^	24.5	11–30
Reported exercise (kcal/week)	1038 (735)	956	0–2250
**DIAGNOSIS**			
Vascular cognitive impairment	13	–	–
Transient ischemic attack	6		–
Coronary artery disease	2	–	–
Cholesterol lowering agent	11	–	–
Hypertension	13	–	–
Diabetes	3		–
**NEUROIMAGING CHARACTERISTICS**			
Intracranial vol. (mL)	1216 (152)	1234	919–1571
Brain parenchymal fraction (%)	79.9 (4.2)	78.6	71.7–86.3
WM vol. (mL)	417 (60)	423	282–512
WMH vol. (mL)	22.7 (16.5)	17.1	2.2–70.6
WMH Fazekas score	4.65 (1.3)^†^	5.0	2–6
CBF -GM (ml/100 g/min)	47.7 (17.5)	46.7	21.6–98.5
CBF-WM (ml/100 g/min)	34.6 (14.2)	35.9	12.5–64.7

† *Non-normal distribution*.

**Figure 2:**
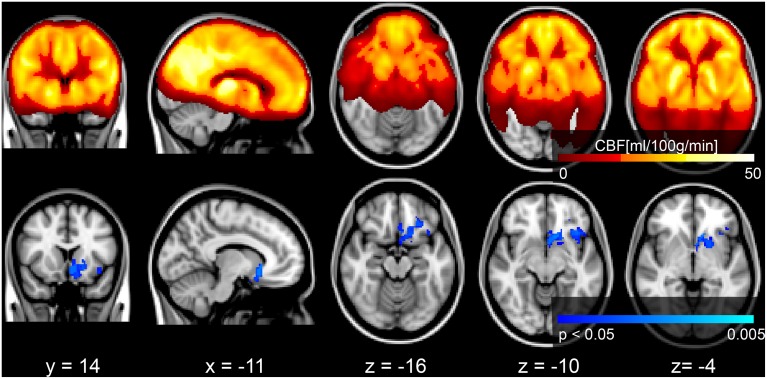
**Top row:** mean CBF image of the group. **Bottom row:** Results of a voxel-wise analysis of CBF vs. WMH volume shows decreased perfusion with increasing disease burden, signified by the blue voxels (*p* < 0.05, corrected for multiple comparisons, age and sex). The gray scale anatomical image is the ICBM-152 template in radiological convention.

**Figure 3:**
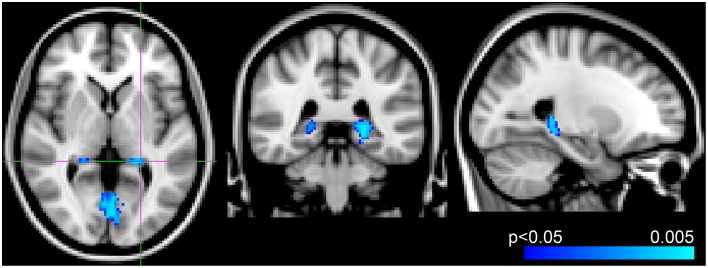
**Results from voxel-wise analysis showing region with reduced gray-matter-estimate correlating with WMH volume overlaid onto ICBM-152 template (blue,** ***p*** **< 0.05, corrected for age and sex)**. Slices at *z* = 2 mm, *y* = −38 mm, *x* = −24 mm from right to left, respectively.

Voxel-wise analysis of the CBF data revealed a negative relationship between WMH volume and CBF in the left anterior putamen, subcallosal, accumbens, anterior caudate, orbital frontal, anterior insula, and frontal pole (*p* < 0.05, corrected for age and sex). Refer to Figure 2 for a map of the significant voxels and Table 2 for MNI coordinates and cluster size. Figure 3 shows the result of the VBM, indicating there was a significant negative relationship between WMH volume and GM estimates in lingual gyrus, intracalcarine, and bilateral hippocampi (*p* < 0.05, corrected, Table 2). Table 2 shows the association results between the regional CBF values and the Fazekas score.

**Table 2:** **Voxel-wise results**.

	**MNI coordinates (mm)**				**Association with Fazekas (rho)**	
	***x***	***y***	***z***	**# voxels**	**rho**	***p*-value**
**CBF**						
(a) Left anterior putamen, subcallosal, accumbens, anterior caudate, orbital frontal	−11	14	−9	496	−0.55	0.006^*^
(b) Left anterior insula, orbital frontal	−37	20	−10	166	−0.49	0.015^*^
(c) Left orbital frontal, frontal pole	−26	32	−15	69	−0.56	0.005^*^
**VBM**						
(d) Medial lingual gyrus, intracalcarine	−3	−69	1	615	−0.42	0.040^*^
(e) Left hippocampus	−21	−37	−3	126	−0.52	0.009^*^
(f) Right hippocampus	21	37	1	29	−0.61	0.002^*^
(g) Medial lingual gyrus	5	−66	−10	17	−0.57	0.004^*^

MNI Coordinates given for center of mass of cluster, with extents >10 voxels. Brain regions listed if more than 20 cluster voxels extend into this region. Post-hoc results show partial correlation between cluster means and Fazekas rating corrected for age and sex (

* *indicates p < 0.05)*.

In the exploratory analysis that compared the CBF and VBM findings, there was a significant partial correlation between CBF and VBM foci for 6 of the 12 comparisons, after Bonferroni correction and adjustment for age and gender (Table 3). Including WMH as an additional covariate made all correlations non-significant (unadjusted *p* > 0.27).

**Table 3:** **Partial correlation of extracted CBF and GM values in ROIs identified by voxel-wise analysis**.

	**Mean CBF in**		
**Mean GM estimate in**	**Left putamen**	**Left insula**	**Frontal ROI**
Lingual, intracalcarine	0.58 (0.03)^*^	0.50 (0.16)	0.61 (0.02)^*^
Left hippocampus	0.60 (0.03)^*^	0.62 (0.014)^*^	0.57 (0.04)^*^
Right hippocampus	0.57 (0.05)^*^	0.56 (0.06)	0.56 (0.06)
Lingual	0.45 (0.31)	0.39 (0.68)	0.49 (0.19)

*Rho is shown corrected for age and sex (p-value in parentheses*,

* *indicates p < 0.05 after Bonferroni correction)*.

## Discussion

In this cross-sectional study we found that WMHs of presumed vascular origin, expressed as the total WMH volume, were significantly associated with intra-group regional differences in gray matter CBF and structure. Our multi-modality approach treated CBF and VBM datasets separately and these analyses identified distinct GM regions that were associated with WMH volume. *Post-hoc* tests provided additional context for our main results. In the first case, use of the WMH visual rating scale, Fazekas score, produced a similar significant finding in ROIs identified by the WMH volume continuous variable. Second, we identified three CBF regions that were associated with WMH and four VBM regions that were associated with WMH. Comparing GM vs. CBF in these regions helped to demonstrate that there were several correlations between CBF and GM estimates.

Although the range in WMH volumes was relatively large in this cohort, from 2.2 to 70.6 mL, the mean lesion load of 23.2mL was moderately large relative to other reports. Some studies state that a WMH volume >1.3 mL (i.e., 0.1% of intracranial volume) is clinically significant (Uh et al., 2010), while others report a threshold for the significant minimum volume as >10 mL (0.8% of intracranial volume) (Boone et al., 1992; Decarli et al., 1995).

### Blood flow finding: reduced flow correlates with WMH volume

Our primary findings were that CBF in the putamen, anterior insula and orbitofrontal cortex were negatively correlated with WMH volume, specifically in the left hemisphere only. In the case of the putamen, this is a region that had reduced CBF in relation to WMH severity (Kawamura et al., 1991). Next, the insula is an important autonomic region, with relevance in WMH to cardiovascular and blood pressure signals (Oppenheimer et al., 1992; Goswami et al., 2012), as well as the insula as a hub in the salience network that pertains to our thoughts, feelings and actions (Menon and Uddin, 2010). Studies of ischemia in the left and right insula have established its role in autonomic heart rate function (Nagai et al., 2010) and a functional study in healthy volunteers has implicated the anterior insular cortex in blood pressure control (Gianaros et al., 2007). We also observed reduced CBF in the orbitofrontal cortex, a region previously shown to have reduced cortical thickness in SVD patients with gait impairments (de Laat et al., 2012). Furthermore, CBF changes in the subcallosal and accumbens areas have been linked to hypertension in humans and mice (Strazielle et al., 2004; Dai et al., 2008).

### Structural finding: decreased hippocampal volume correlates with WMH volume

We observed a negative association between WMH and VBM-derived hippocampal volume, which adds to the body of work that has focused primarily on the hippocampus. For instance, Eckerstrom et al. report a hippocampus and WMH volume association in the upper quartile of their cohort (i.e., >11 mL WMH volume) (Eckerstrom et al., 2011), whereas 1.5 mL WMH burden has been reported as a minimum threshold to contribute to global GM atrophy (Kloppenborg et al., 2012). Despite our modest sample size, we identified additional GM regions that were associated with WMH volume, namely the lingual gyrus and the intracalcarine, which are reported less in the literature (Seo et al., 2010). Our VBM findings may have benefitted by our novel approach of excluding WMH voxels that could be misclassified as GM on T1-weighted images.

The hippocampus is a brain region that is vulnerable to global hypoperfusion (Knoops et al., 2009) and pathological studies have shown that impaired microvascular perfusion can trigger a cascade of events that ultimately result in GM atrophy (Jellinger and Attems, 2007). In addition, there is growing evidence that vascular lesions contribute to dementia (Debette and Markus, 2010). Our voxel-wise CBF and VBM findings support the idea of a shared vascular etiology between WM and GM abnormalities. The next step in this research could be to investigate these findings in the context of memory performance.

### Study design: strengths

This study used a multimodal neuroimaging approach that benefited from a number of factors. First, the multi-modality WMH, CBF, and GM information was used to establish a more comprehensive understanding of how SVD impacts GM and WM regions remote from the lesion locations. Second, robust and automated procedures were used to measure WMH volume and GM estimates, which had the advantage of producing reliable summary metrics for group-level analysis.

### Study design: limitations

The only cognitive test performed in this study was the MoCA, which was used as a screening tool. Thus, we were unable to investigate associations between brain measures and cognition. We collected binary values for clinical diagnosis of risk factors and were thus unable to test for associations across a gradient of potential risk, such as WMH and blood pressure. Nevertheless, our significant CBF results provide support for the theory of an ischemic etiology in SVD (Brickman et al., 2009). Our sample size was small, which precluded our ability to perform analyses on additional patient differences, for example, regional WMH distribution (Tullberg et al., 2004) or vascular risk factors. Although CBF and VBM analyses were conducted on images with the same voxel dimensions, i.e., VBM used down-sampled images, we did not explicitly account for partial volume effects on the CBF results. Furthermore, the ASL was performed at a single PLD, which does not permit arterial transit time mapping, which may be important in this cohort since our choice of PLD may have contributed to macrovascular artifact. Finally, we did not perform carotid doppler ultrasound to rule out large artery disease, instead relying on self-report and chart notes. This could also contribute to macrovascular artifact as a result of prolonged arterial transit times.

## Conclusion

This study demonstrates that WMH in periventricular and deep white matter are associated with decreased gray matter CBF and structural profiles in regions that are remote from the WMH lesions. The etiology of WMH remains a topic of intense research (Thompson and Hakim, 2009; Debette and Markus, 2010; Gibson et al., 2010; Uh et al., 2010; Ramirez et al., 2011; Makedonov et al., 2013a; van der Holst et al., 2013; Wardlaw et al., 2013) with one prevailing view that the lesions are caused by underlying vascular insufficiency (Brickman et al., 2009; Makedonov et al., 2013b; Wardlaw et al., 2013). A number of studies show that hypertension, diabetes, obesity and smoking are risk factors for developing WMH of presumed vascular origin, as discussed in a recent review (Appelman et al., 2009). In this study, we found a negative association between WMH volume and CBF in frontal (i.e., insula and orbitofrontal regions) and the basal ganglia (i.e., putamen) but not the hippocampus. The VBM results showed a negative association with WMH volume in the bilateral hippocampus and occipital regions. Longitudinal non-invasive neuroimaging techniques, such as the CBF and VBM approaches presented here, may help to explain how and why the progression of WMH lesion volume contributes to cognitive changes.

### Conflict of interest statement

The authors declare that the research was conducted in the absence of any commercial or financial relationships that could be construed as a potential conflict of interest.
